# Concurrent Estimation of Clopidogrel Bisulfate and Aspirin in Tablets by Validated RP-HPLC Method

**DOI:** 10.4103/0250-474X.45414

**Published:** 2008

**Authors:** P. K. Shrivastava, P. K. Basniwal, Deepti Jain, S. K. Shrivastava

**Affiliations:** Department of Pharmaceutics, Institute of Technology, Banaras Hindu University, Varanasi-221 005, India; 1 School of Pharmaceutical Sciences, Rajiv Gandhi Proudyogiki Vishwavidyalaya, Airport By pass Road, Gandhi Nagar, Bhopal-462 036, India; 2 L. B. S. College of Pharmacy, Tilak Nagar, Jaipur-302 004, India

**Keywords:** Reverse phase High Performance Liquid Chromatography (RP-HPLC), clopidogrel, aspirin, method validation, ICH guidelines

## Abstract

A simple, rapid, precise RP-HPLC method was developed for simultaneous estimation of aspirin and clopidogrel bisulphate in tablet dosage form used in the treatment of cardiovascular diseases. To achieve the maximum resolution, acetonitrile:50 mM potassium dihydrogen phosphate buffer:methanol, solution pH adjusted to 3, in the ratio 50:30:20; v/v was selected as mobile phase. This mixture was found to be appropriate allowing good separation of both the components at a flow rate of 1.5 ml/min and detection wavelength 240 nm. In these conditions clopidogrel bisulfate and aspirin were eluated at the 7.47 and 2.2 min. The linearity was found in the concentration range 1.5-7.5 and 3.5-15.0 μg/ml, respectively. All the analytical validation parameters were determined and found with in the limit as per ICH guideline, which indicates the validity of method.

Clopidogrel bisulphate (CPS) is methyl-2-chlorophenyl-(4,5,6,7-tertrahydrothieno[3,2-c] pyridine-5yl)acetate bisulphate and aspirin (ASP) is 2-acetoxy benzoic acid used in the treatment of cardiovascular diseases. Clopidogrel is used as platelet inhibitor and aspirin as a cyclooxygenase inhibitor^1^,^2^. Monographs of various pharmacopoeias describe the assay of aspirin. Literature review reveals few HPLC methods reported for estimation of both the drug as single component in different dosage form, but no method has yet been reported for analysis of these drugs in multi component dosage form^3^–^9^. The objective of present communication was to develop simple, rapid and precise RP-HPLC method for concurrent estimation of clopidogrel bisulphate and aspirin in tablet dosage form.

Clopidogrel bisulphate and aspirin were gift samples received from Aristo Pharmaceutical, Mandideep, India. HPLC grade acetonitrile and methanol were obtained from Merck (India) Limited. Analytical grade potassium dihydrogen phosphate and orthophosphoric acid were purchased from S. D. Fine Chemicals, Mumbai. The tablet of clopidogrel bisulphate and aspirin (Clopivas-AP-150, Cipla Ltd, Mumbai) were procured from the market in the strength of 75 mg and 150 mg, respectively.

The HPLC system consisted of a solvent delivery module LC-10ATvp Shimadzu Liquid chromatograph pump equipped with 20 μl loop and model SPD M10Avp Shimadzu UV/VIS diode array detector. Integration was achieved by using the software LC-10^10^. Separation was carried out on a Phenomenex, (250×4.6mm) Luna 5μ C-18 (2) 100A column. The chromatographic analysis was performed at ambient temperature. The mobile phase consisted of the mixture of solvents, acetonitrile:50 mM potassium dihydrogen phosphate buffer:methanol, solution pH adjusted to 3.0 with orthophosphoric acid, in the ratio 50:30:20; v/v. The prepared mobile phase was filtered through a Millipore 0.45 μm membrane filter and ultrasonically degassed prior to use. The detection wavelength was set at 240 nm and the peak area was recorded using chromatographic data system. The flow rate and run time was set to 1.5 ml/min and 10 min, respectively. The representative chromatogram is given in fig. 1. All the system suitability parameters capacity factor, plate number, tailing factor, retention time, and resolution were optimized by freshly prepared standard solution of CPS (3.0 μg/ml) and ASP (15.0 μg/ml).

**Fig 1:**
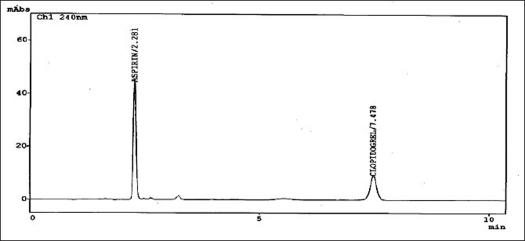
Representative chromatogram of aspirin and clopidogrel bisulfate mixed standard at detection wavelength 240nm.

To develop a suitable method accurately weighed CPS and ASP were dissolved in mobile phase (ACN:buffer:meOH (5:3:2 v/v) to obtain solution of 1.5, 3.0, 4.5, 6.0 and 7.5 μg/ml for CSP and 3.0, 6.0, 9.0, 12.0 and 15.0 μg/ml for ASP. Before the HPLC injection the solution were filter through 0.45 micron HPLC filter. Peak area under the curve of mixed standard were observed and plotted against respective concentration and linearity was observed in the range of 0.0-7.5 μg/ml for CPS and 0.0-15.0 μg/ml for ASP at the 240 nm.

The LC Method for simultaneous estimation of clopidogrel bisulfate with aspirin combination was extensively validated as per ICH guideline for analytical method validation, for linearity, range, accuracy, precision (repeatability and intermediate precision)^11^. For linearity, series of dilutions were prepared and response ratios were determined respectively. The range was determined by preparing a series of dilutions from 80% to 120% of test concentration in six replicate. By recovery studies in which known amounts of standard drug were added to the previously analysed tablet sample and mixture were analysed by the proposed method, accuracy of methods was determined. Precision was studied for repeatability and intermediate precision (days, and analysts).

For analysis of tablet sample (Clopivos-AP-150 in the strength 75:150 mg, Cipla limited, Mumbai), twenty tablets were accurately weighed and average weight of the tablets was determined. The tablets were powdered and powder equivalent to 75 mg of CPS and 150 mg of ASP were accurately weighed and dissolved in mobile phase and volume was made-up to 10 ml. Resulted solution was sonicated for 10 min and filtered through Whatman filter paper no. 42. Solutions of different concentrations were prepared by serial dilution technique as per standard preparation and were subjected for analysis. The optimized chromatographic conditions employed (system suitability parameter) to obtain best resolution between two compounds are shown in Table 1.

**TABLE 1:** RESULTS OF SYSTEM SUITABILITY PARAMETER FOR CLOPIDOGREL BISULFATE AND ASPIRIN

System suitability parameter	CPS	ASP	SD*		RSD*	
				
			CPS	ASP	CPS	ASP
Retention time (min)	7.476	2.2036	0.00743	0.00320	0.00099	0.00146
Capacity Factor	2.274	-	0.00547	-	0.00240	-
Plate Number	12051	4441.8	36.50	29.73	0.00302	0.00669
Tailing Factor	1.048	1.262	0.0192	0.0044	0.0183	0.00348
Resolution	26.042	-	0.320	-	0.0122	-

* Maximum values of SD and RSD of six replicates

For standard drug, linearity was observed by linear regression equation method. The equation were, AUC=17386.4X− 459.636 for CPS and AUC=7432.58X+65.78 for ASP and correlation coefficient for both drugs were found to be more than 0.999, indicating good linearity.

The LC Method was extensively validated for simultaneous estimation of CPS with ASP combination using linearity, range, accuracy and precision. All these analytical validation parameters were observed and the RSD was found to be less than one, which indicates the validity of method.

The concentration of CPS and ASP estimated in the tablet was found to be in the range 99.33-100.66% and 99.33-101.33%, while the standard deviation values obtained from replicate analysis of tablet were found to be in the range of 0.0152-0.0208 and 0.0321-0.0585, respectively, which indicates satisfactory applicability and reproducibility of the method (Table 2). The standard deviations of recovery studies were also found 0.0670 for CPS and 0.0679 for ASP

**TABLE 2:** RESULTS OF TABLET ANALYSIS FOR CLOPIDOGREL BISULFATE AND ASPIRIN

Parameter	Limit*	
	
	CPS	ASP
% Found	99.33-100.66	99.33-101.33
SD	0.0152-0.0208	0.0321-0.0585
RSD	0.0027-0.0102	0.0038-0.0107

* Maximum and minimum values of three different concentration level in three replicates.
